# Tumorigenic Factor CRIPTO-1 Is Immunolocalized in Extravillous Cytotrophoblast in Placenta Creta

**DOI:** 10.1155/2014/892856

**Published:** 2014-08-06

**Authors:** Carla Letícia Bandeira, Alexandre Urban Borbely, Rossana Pulcineli Vieira Francisco, Regina Schultz, Marcelo Zugaib, Estela Bevilacqua

**Affiliations:** ^1^Department of Cell and Developmental Biology, Institute of Biomedical Sciences, University of São Paulo, Avenida Professor Lineu Prestes, No. 1524, Cidade Universitária, 05508-900 São Paulo, SP, Brazil; ^2^Department of Obstetrics, Clinics Hospital, School of Medicine, University of São Paulo, Avenida Dr. Enéas de Carvalho Aguiar, No. 255, Cerqueira César, 05403-000 São Paulo, SP, Brazil; ^3^Division of Pathology, Clinics Hospital, School of Medicine, University of São Paulo, Avenida Dr. Enéas de Carvalho Aguiar, No. 255, Cerqueira César, 05403-000 São Paulo, SP, Brazil

## Abstract

CRIPTO-(CR)1 is a protein associated with tumorigenesis and metastasis. Here we demonstrate that CR-1 expression in normal and creta placentas is associated with various degrees of uterine invasion. Cytokeratin (CK) and CR-1 protein expression was visualized by immunohistochemical staining of formalin-fixed, paraffin-embedded placental specimens (control placentas, *n* = 9; accreta, *n* = 6; increta, *n* = 10; percreta, *n* = 15). The pattern of extravillous trophoblast (EVT) cell morphology was distinctive in creta placentas: densely-compacted cell columns and large star-shaped cells with a typically migratory phenotype, not common among third trimester control placentas. Quantification revealed higher CR-1 immunoreactivities in accreta (*P* = 0.001), increta (*P* = 0.0002), and percreta placentas (*P* = 0.001) than in controls. In contrast to controls, there was a significant positive relationship between CR-1 and CK reactivity in all creta placentas (accreta, *P* = 0.02; increta, *P* = 0.0001, and percreta, *P* = 0.025). This study demonstrated CR-1 expression in the placental bed, its increased expression in creta placentas, and EVT cells as the main CR-1-producing cell type. Morphological examination revealed an immature and invasive trophoblast profile in creta placentas, suggesting impairment of the trophoblast differentiation pathway. These findings provide important new insights into the pathophysiology of abnormal creta placentation and its gestational consequences.

## 1. Introduction

Abnormal placentation is one of the most common pregnancy complications, and placenta creta is a common concomitant; it is closely associated with the need for hysterectomy and its consequences can lead to maternal death [1–5]. Placenta creta was originally diagnosed in 1930 [6] and its incidence has increased over the years (1 : 2,510 in 1980 [7] and 1 : 533 in 2002 [8]), closely associated with the incidence of placenta previa and increasing number of Cesarean sections [8–12].

Total or partial absence of decidual tissue reaction is a common histological characteristic of placenta creta, so the placental villi insert directly into the myometrium [13–15]. The pathogenesis of placenta creta remains to be elucidated, but evidence indicates that it is primarily a maternal disease associated with decidual deficiency, which can be due to uterine scarring and with secondary consequences for the regulatory mechanisms of trophoblast invasion and function [14, 16, 17].

Considered a fully iatrogenic pathology [18], placenta creta is currently classified according to the depth of abnormal adhesion and invasion of the chorionic villi to the myometrium in the absence/deficiency of decidualization, taking into consideration whether the placental insertion is superficial or deep and whether or not it transcends the serous layer to reach adjacent structures such as the bladder and ureters [6, 13, 14, 19]. These descriptions characterize the subtypes of creta placentas as accreta, increta and percreta, respectively [14–16]. Abnormal invasion into the deeper layers of the myometrium is accompanied by a distinctive placental neovascularization. In consequence, exacerbated vascular remodeling usually reaches the radial, arcuate and parametrial arteries, increasing the caliber of these vessels, which become barely capable of homeostatic response after placental abruption [20–23].

The factors responsible for invasive placental activity during normal and pathological placentation are not completely understood at the cellular level. Impairment of regulatory signaling between these cells and the cellular and noncellular decidual components has been strongly proposed, along with modulation of the expression of for example, growth factors, hormones, cytokines, adhesion molecules, and oncogenes by the components of the maternal-fetal interface [23–26].

Data obtained through cDNA microarray analysis of mouse placentas have demonstrated that the CRIPTO-1 oncogene is highly expressed at the maternal-fetal interface [27]. CRIPTO-1 is a member of the epidermal growth factor-CRIPTO-1/FRL-1/Cryptic (EGF/CFC) family, abundantly expressed in embryonic stem cells and tumor cells [28, 29]. Furthermore, it is overexpressed in various primary human carcinomas (breast, lung, colon, gastric, pancreas, ovary, cervix, endometrium, and testis) [30, 31], suggesting a role in tumorigenesis, particularly in angiogenesis and invasiveness [28, 31].

Considering that creta placentas are characterized by a prominent deviation of villous invasion, we hypothesize that CRIPTO-1 is expressed by the invasive placental population and we examine its expression at the maternal-fetal interface using immunohistochemistry. Creta placentas of various degrees and placentas from healthy gestations were quantitatively and qualitatively analyzed and compared.

## 2. Materials and Methods

### 2.1. Sample Collection

Paraffin blocks of formalin-fixed placenta samples were selected from the archives of the Division of Pathology at Clinics Hospital, School of Medicine, University of São Paulo. They included six maternal-fetal interface areas from placenta accreta (from 36 weeks of gestation), 10 maternal-fetal interface areas from placenta increta, and 15 samples from placenta percreta (37-38 weeks of gestation) obtained from immediate postpartum hysterectomy. Control (non-creta) cases consisted of nine third trimester placentas (*n* = 3, 36 gestation weeks [gw], and *n* = 6, 38 gw) from elective cesareans from healthy mothers and fetuses (without chronic hypertension, renal disease, vascular disease, infection, fetal anomalies, or any other pregnancy complications). Maternal risk factors for placentas creta are summarized in Table 1.

The placentas were conventionally diagnosed as accreta (superficially implanted), increta (within the myometrium), and percreta (through the myometrium) by morphological examination using clear evidence of loss of decidua and the degree of myometrial adhesion as criteria. The study was approved by the Ethics Committee for Human Research at the School of Medicine, University of São Paulo.

Because the gestational age differed between the control (healthy) and pathological (accreta, increta, and percreta) placenta groups, respective gestational age-matched groups were used as controls (placentas of 36 gw for placenta accreta and placentas of 38 gw for placenta increta and percreta).

### 2.2. Immunohistochemistry

The paraffin blocks were semiserially sectioned at 5 *μ*m intervals and mounted on slides and processed for immunohistochemical staining. Standard conditions included immunostaining of three separate groups subjected to the same experimental conditions: (i) a group containing the accreta placentas and age-matched normal placentas (36 gw), (ii) a group containing increta and percreta placentas and the age-matched controls (38 gw), and (iii) a group comprising healthy placentas from 36 and 38 gw. The primary antibodies were rabbit polyclonal IgGs against human CRIPTO-1 protein (Santa Cruz Biotechnology, Santa Cruz, CA), cytokeratin LMW, and vimentin (Cell Marque Corporation, CA, EUA), respectively, diluted at 1 : 100, 1 : 350, and 1 : 100. Goat anti-rabbit and goat anti-mouse IgG (KPL, Kirkegaard & Perry Laboratories, Inc, USA) were used as second antibodies at 1 : 100 dilutions. The antigens in the sections were visualized using a DAB substrate kit for peroxidase (Vector Laboratories Inc., CA). Slides were counterstained with Mayer's hematoxylin. Sections from each placental group were used as negative controls with the primary antibody replaced with Tris-buffered saline or nonimmune rabbit serum.

### 2.3. Quantitative and Statistical Analysis

Images of the immunoreactions were acquired and captured using an Axioskop 2 Optical Microscope equipped with Axio Vision 4.7 software (Carl Zeiss MicroImaging GmbH, Jena, Germany). Quantification was performed on images captured using a × 10 objective, 1,388 × 1,040 pixels, and a resolution of four pixels/*μ*m^2^. Five images from each slide from five paraffin blocks randomly selected for each group were captured, resulting in 25 images per group for comparison.

Using computer-assisted image analysis (ImageJ, NIH, USA), the density of cytokeratin and CRIPTO-1 immunoreactivity was measured in each field of each slice and averaged. The microscopic area was calculated as 90.220 *μ*m^2^ and the results were recorded as pixels/*μ*m^2^. The ratios of CRIPTO-1 to cytokeratin were calculated in each field and the means were statistically compared.

The package PRISM Graph Pad for PC (version 5.0) was used for statistical analyses. A nonparametric one-way ANOVA (Kruskal-Wallis) with Dunn's multiple comparison posttest was used for all-group comparisons. Results were expressed as mean ± SD. The level of significance was set at *P* < 0.05.

## 3. Results

To assess similar areas of the maternal-fetal interface among the samples, each tissue was stained with hematoxylin/eosin (HE) (data not shown) and further characterized by vimentin, cytokeratin, and CRIPTO-1 immunostaining.

### 3.1. Placental Bed in Healthy Placentas

Deciduae of placentas from healthy gestations obtained at 36 and 38 weeks of gestation were characterized by a face in which villi were attached contiguously to connective tissue containing different cell populations. This region, which denominated the basal plaque, comprises decidual cells, leukocytes, components of the endometrial vasculature, and cytotrophoblast cells represented as interstitial and endovascular extravillous cells dispersed in a distinctive extracellular matrix. The decidual vasculature was represented by a net of distended lumen capillaries and more structured vessels in the deeper areas of the decidua. No morphological differences could be found among samples from these three gestational periods (data not shown).

**Figure 1:**
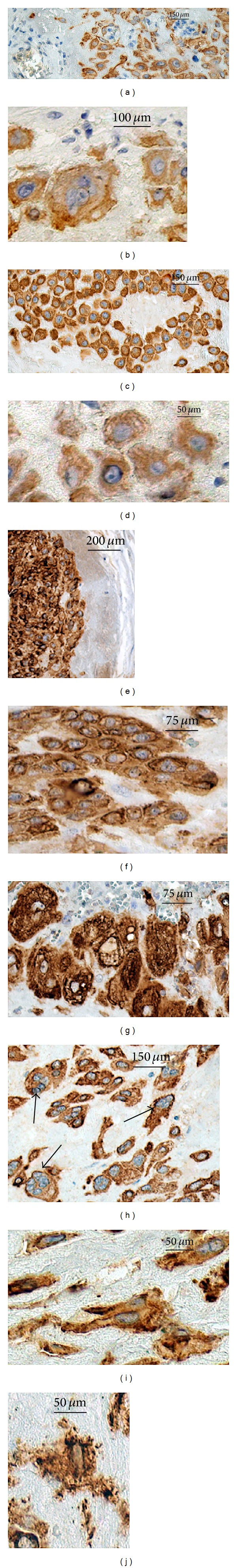
Histological characterization of healthy and creta placentas. Representative histological sections revealing cytokeratin-reactive cells in the placental bed of (a–d) term healthy placenta (36 gw) and (e–j) creta placentas (accreta: (e), (g), and (i); percreta: (f), (h), and (j)). Note cytokeratin-reactive cells (brownish color) disposed in healthy placentas as a group of large, polygonal cells that apparently do not maintain contact with other cells (a–d). In (b) note a typical multinucleate trophoblast cell. (e–j) In creta placenta samples, cytokeratin-positive cells are organized as group of compact cells (e–g) or as isolated star-shaped cells (i-j). Arrows indicate multinucleate trophoblast cells (h). Immunoperoxidase, Mayer's hematoxylin counterstaining.

**Figure 2:**
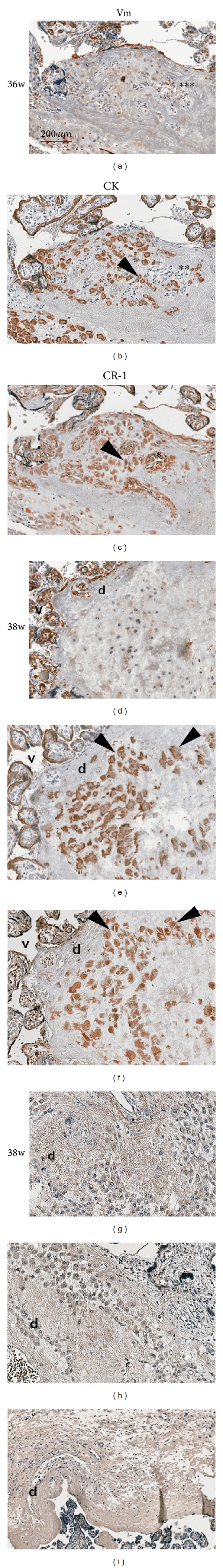
Expression of CRIPTO-1 and cell markers in healthy placentas. (A) Representative histological sections demonstrating the immunolocalization of vimentin (Vm: a, d), cytokeratin (CK: b, e), and CRIPTO-1 (CR-1: c, f) in placental beds from healthy placentas at 36 (a–c) and 38 (d–f) weeks of gestation. Arrowheads indicate cells reactive to cytokeratin and CRIPTO-1 in semiserial histological sections. Asterisks identify vascular areas stained for vimentin and CRIPTO-1 (a, c), but not for cytokeratin (b). (g–i) Negative control of the immunohistochemistry reactions in which the respective primary antibody has been omitted. Immunoperoxidase, Mayer's hematoxylin counterstaining. Bar in (a) = 200 *μ*m in all figures.

Immunolocalization of cytokeratin and vimentin intermediate filaments in tissue sections of the basal plaque allowed us to characterize the populations of trophoblast (Figures 1-2) and nontrophoblast (Figure 2) cells.

Extravillous cytokeratin-positive cytotrophoblast cells were scattered throughout the decidua (Figures 1(a)–1(d)). Although this antigen is expressed by all cells of epithelial origin, the absence of maternal luminal/glandular epithelium during the final stages of gestation usually makes cytokeratin reactivity a suitable marker for locating extravillous trophoblast cells among the other decidual cells (decidual cells, stromal cells, macrophages, lymphocytes, and natural killer cells are cytokeratin-negative). Cytokeratin-reactive cells comprised a variable proportion of large-dimension polygonal cells (Figures 1(a)–1(d)) and occasional multinucleated cells dispersed in diffuse sheets or nests (Figure 1(b)). These cells displayed euchromatic nuclei with prominent nucleoli. Cytokeratin-positive cells lining the maternal vessels were also common (Figure 1(a)), mainly in deeper areas of the decidua.

Cells exhibiting morphological characteristics similar to CK-reactive extravillous cytotrophoblast cells (Figures 2(b) and 2(e)) were the main intensely CRIPTO-1-immunoreactive cell type in decidua (Figures 2(c) and 2(f)) at both 36 and 38 gw. Some endothelial cells in the deeper portions of the decidua were also CRIPTO-1 immunoreactive (Figures 2(a) and 2(c)).

**Figure 3:**
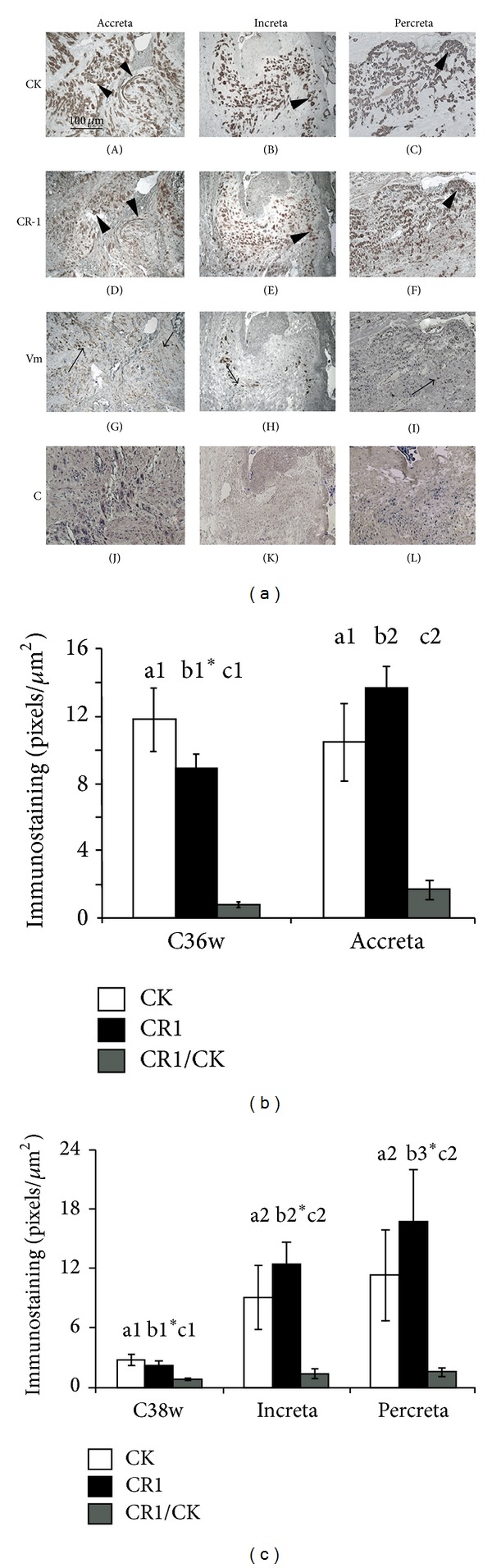
Expression of CRIPTO-1 and cell markers in creta placentas. (a) Representative histological sections demonstrating immunolocalization of cytokeratin (CK: A–C), CRIPTO-1 (CR-1: D–F), and vimentin (Vm: G–I) in representative cases of accreta (A, D, G, and J), increta (B, E, H, and K) and percreta (C, F, I, and L) placentas. The arrowheads indicate cells reactive to cytokeratin and CRIPTO-1 in semiserial histological sections. Arrows depict vimentin-positive cells. ((c), J–L) Negative control of the immunohistochemistry reactions in which the respective primary antibody has been omitted. Immunoperoxidase, Mayer's hematoxylin counterstaining. Bar in ((a)(A)) = 100 *μ*m in all figures. (b-c) Quantification of the immunoreactivity (pixels/*μ*m^2^) for cytokeratin (CK) and CRIPTO-1 (CR-1) proteins at the maternal-fetal interface in placentas from healthy mothers (gestation week 36) and accreta placentas (b) and of healthy placentas (gestation week 38) and increta and percreta placentas (c). Different superscript letters above the bars indicate the group statistically analyzed; means with different numbers are significantly different, *P* < 0.05, whereas means with similar numbers do not differ. Asterisks indicate significant differences in relation to CK in the same group (*P* < 0.05). The results of the analysis are given in the text.

Quantification of cytokeratin (CK)- and CRIPTO-1 (CR-1)-reactive cells in the placental bed from healthy gestations (Figures 3(b) and 3(c)) revealed a significant difference between CK and CR-1 immunointensities at gestation weeks 36 (11.85 ± 1.89 and 8.92 ± 0.78, resp., *P* = 0.001) and 38 (2.75 ± 0.43 and 2.22 ± 0.37, resp., *P* = 0.002). However, there was no significant difference in the CR-1/CK ratio (36 w, 0.77 ± 0.18; 38 w, 0.81 ± 0.16).

### 3.2. Maternal-Fetal Interface Areas in Creta Placentas

The maternal-fetal interface in creta placentas (Figure 3) was characterized by endometrial/myometrial/perimetrial hemorrhage, leukocyte infiltration, areas of leakage and necrosis, and almost total absence of decidual cells. The examinations were mainly performed on the transitional area between the atrophic endometrium and myometrium in accreta placenta and in the myometrium in increta and percreta placentas.

In all specimens, the vimentin antibody stained endothelial cells, leukocytes, and fibroblasts (Figures 3(a), (G)–(I)). Cytokeratin-positive cytotrophoblast cells permeated muscle cells and were morphologically different from those found in healthy placentas. They were either organized as a compact group of histologically and immunophenotypically homogenous cells (resembling tightly packed colonies; Figures 1(e)–1(g)) or were sparsely distributed (Figures 1(h)–1(j)). Isolated cells displayed migratory characteristics, exhibiting star-shaped cytoplasm and long projections (Figures 1(h)–1(j)).

The increta placentas had similar characteristics although the cytokeratin-reactive cytotrophoblast invasion reached deeper layers of the myometrium. In placenta percreta, cytokeratin-reactive cells were also found lining the perimetrium. Cytokeratin-reactive cell aggregates often surrounded and/or lined the uterine vessels.

CRIPTO-1 colocalized with most of the large cytokeratin-reactive cells in the placental bed (Figure 3(a)) at all levels in the creta placentas here analyzed. However, CRIPTO-1 expression was not exclusive to this cell population. Endothelial and myometrial cells were also immunoreactive to the anti-CRIPTO-1 antibody.

Quantification of cytokeratin- and CRIPTO-1-reactive cells in the placental bed demonstrated significantly higher immunointensity for CR-1 (13.67 ± 1.55, *P* = 0.001) and for the ratio CR-1/CK (1.61 ± 0.53, *P* = 0.02), but not for CK (10.46 ± 4.97), in accreta placentas than in the age-matched control group (Figure 3(b)).

The intensity of CR-1-reactive cells was higher in increta and percreta placentas (Figure 3(c)) than in the respective CK-reactive cell population (12.54 ± 2 versus 9.09 ± 3.11, *P* = 0.008 and 18.22 ± 4.26, *P* = 0.04) and higher than in the age-matched control (*P* < 0.05). The CR-1/CK ratio was approximately 2-fold higher in the pathological placentas (increta, 1.47 ± 0.35 and percreta, 1.65 ± 0.54, *P* < 0.05) than in the controls.

## 4. Discussion

Abnormal placentation is one of the most common pregnancy complications, and creta placentas appear extensively among them; they are closely associated with the need for hysterectomy with consequences that can lead to maternal death [1–5]. Creta placentas are becoming more common, with their incidence increasing over the years (1 : 2,510 in 1980 [7] and 1 : 533 in 2002 [8]). Several factors have been implicated in this augmentation, primarily: the increasing incidence of placenta previa, the increasing proportion of deliveries by caesarean, and the rising maternal age at delivery (>35 years) [8–12, 16, 18]. In this study our selected pathological groups exhibited many of the risk factors, singly or in association; all had some kind of prior uterine surgery and almost all (60–80%) had cesarean sections and placenta previa.

Despite the factors or combination of factors that increase the risk for placenta creta, its exact etiology is still unknown. In the present study we found, using immunohistochemistry, increased CRIPTO-1 expression in the term placental bed and in creta placentas exhibiting different degrees of abnormal implantation relative to normal placentation. Moreover, we described for the first time that this expression is particularly associated with cells morphologically characterized as extravillous cytotrophoblast cells.

In the placental bed, CRIPTO-1 expression colocalized with cytokeratin-reactive cells in the semiserial sections, suggesting that extravillous cytotrophoblast cells are the main CRIPTO-producers at this site. We believe that our findings could underscore the specific roles of trophoblast cells at the maternal-fetal interface. CRIPTO-1 signaling within tumor cells has previously been demonstrated to modulate cellular growth, survival, and invasion in several human cancers [30, 32, 33], and this could be especially relevant to the biology of trophoblast cells. In particular, extravillous cytotrophoblast cells are nonproliferative and exhibit a low apoptotic index during the late stages of gestation, which suggests the hypothesis that CRIPTO-1 contributes to trophoblast invasiveness or cell survival mechanisms [34]. As a survival factor, CRIPTO-1 acts through a phosphoinositol-3 kinase (PI3 K-) dependent signaling pathway involving AKT and GSK-3*β* [35], which could be an active mechanism in trophoblast cells [36]. Further studies are required to elucidate the mechanisms underlying CRIPTO-dependent responses in trophoblast cells.

The ratio of CRIPTO-1/cytokeratin reactive cells in healthy placentas indicates that not all CK+ trophoblast cells express this factor; however, this relationship is significantly different in creta placentas. In line with this, we found CRIPTO-1 to be expressed in these placentas, extrapolating the reactivity to the trophoblast cell population, and also in the endothelial and myometrial cells. CRIPTO-1 was abundant in percreta and accreta placentas and less abundant in increta placenta. These data suggest a relationship between CRIPTO-1 and the overall degree of placental invasiveness, in which trophoblast cells are of pivotal importance. Moreover, this finding adds one more item to the list of similarities between trophoblast and cancer cells.

Our quantitative data also highlight variations in the CK-reactive cell population in the placental bed during the last weeks of a healthy gestation, which is corroborated by previous studies [14] and the proportionality between CK and CRIPTO-1 reactivities during those final gestational stages. Interestingly, our previous study demonstrated that the extravillous trophoblast cells retained some capacity for migration and invasion, though it was less than in first trimester placentas [37]. These findings reinforce the conclusion that CRIPTO-1, mostly expressed in potentially invasive cells, is associated with this cellular activity.

Our quantitative analysis demonstrated increased intensity of CK+ cells in increta and percreta placental beds, perhaps because there are more trophoblast cells. Few studies have addressed this possibility. Ki-67 staining is rarely seen in the extravillous trophoblast, indicating low proliferation [38], though a significantly thicker layer of implantation-site intermediate trophoblast and more extravillous cytotrophoblast cells have also been reported [39]. An increased number of cells could result from a critical imbalance between trophoblast cell proliferation and death, leading to the accumulation of this specific cell population over time, and this could explain our results at least in part. However, another possible explanation is the distribution of trophoblast cells in these pathologies. The absence of decidua could lead to an atypical invasion process, in which trophoblast cells would form a more compact front of invasion [39] unlike the situation in a normal pregnancy, when these cells are better distributed along the endometrial maternal-fetal interface.

Our histological study also revealed different patterns of CK+ trophoblast cell distribution and morphology in creta placentas. Overall, these placentas had cells organized as confluent groups, resembling epithelium-like cells growing cohesively as compact islands. These arrangements are similar to the cytotrophoblast cell column organization found during the first trimester in normal placentas [14], but not in the third trimester. The cohesive arrangement of these cells could suggest a coordinate organization of daughter cells following division, though they could also result from post-division aggregations. In any case, cell-cell interactions could be supported by the expression of distinctive adhesive proteins or specific intercellular junctions, which is atypical behavior for the third trimester extravillous cytotrophoblast. Given the severity and outcome complications of these pathologies, more detailed studies should be conducted to clarify the behavior of these cells at cellular, subcellular, and molecular levels.

Cells from accreta placentas also include multinucleate giant cells and cells with invasive morphological characteristics. Large star-shaped cells presenting long projections distributed among the myometrial fibers seem to replace the polygonal cells found in normal placentas. Maintenance of the invasive phenotype in accreta placentas was suggested by Kim et al. [39] and again reveals characteristics normally found during very early pregnancy.

In summary, the morphological features of the extravillous cell population in the placental bed of accreta placentas suggest that the differentiation characteristics of earlier stages have been maintained. Under this perspective, regardless of the factors contributing to this invasive profile (absence of decidual regulatory factors, e.g.), it could partially explain the abnormal invasion by creta placentas.

The mechanisms underlying the expression of CR-1 in placentas and particularly in extravillous trophoblast cells are still to be studied. However, experimental studies using tumor cells have demonstrated that CR-1 is closely regulated by transforming growth factor (TGF)-*β* superfamily members, and particularly by TGF-*β*1 and bone morphogenetic protein (BMP)-4, both expressed by endometrial cells [40]. TGF-*β*1 upregulates CR-1 expression, whereas BMP-4 downregulates it [41]. Therefore, control of the balance between these two factors is relevant to CR-1 expression and activity and could be markedly changed by endometrial impairment with absence/defect of decidua, as seen in creta placentas.

Taking these findings together, we suggest that CRIPTO-1 is part of the mechanism that leads to abnormal placental development. Furthermore, these data provide important new insights into the pathophysiology of creta placentation, affording possibilities for studying its underlying mechanisms and gestational consequences.

## Figures and Tables

**Table 1:** Maternal risk factors for placentas creta incidence.

	Accreta	Increta	Percreta	Normal
	*n* = 6	*n* = 10	*n* = 15	*n* = 9
Prior Gestation %				
(number of gestations)				
(1-2)	33	20	40	78
(≥3)	67	80	60	11
Prior uterine surgery %∗	100	100	100	89
C-section %	83	90	93	89
(number of surgeries∗)	(1-2)	(2–5)	(1–4)	(1-2)
Age ≥ 35 yr %	50	40	33	22
Placenta praevia %	66	70	80	0
Praevia + C-section %	66	60	80	0
Prior abortion %	66	70	33	0
(range)	(1–3)	(1–4)	(1)	0

*Including curettage.
